# Supplementary material for: The transforming dairy sector in Ethiopia
Source: PLoS One. 2020 Aug 13;15(8):e0237456. doi: 10.1371/journal.pone.0237456 (PMC7425916; doi:10.1371/journal.pone.0237456)
Supplement: Attachment — Submitted filename: Plos One review.docx [file pone.0237456.s001.docx]

This study gives in-depth analysis of structural transformation in the Ethiopian dairy sector, focusing on Addis Ababa, the capital of Ethiopia. The study found that dairy consumption in the metropolitan area of Addis Ababa has increase between 2005 and 2016 but still relatively low compare to average global consumption. Milk yield has been increasing in the Urban area over the decade while rural farmers have been experiencing almost stagnant milk yield over the decade. One of the main determinants of productivity gain in the urban farms is the adoption of new breeds of cow.

I have read the paper very well and I think it is suitable for publication in this journal. Below are my comments.

**Major:**

1. The authors should briefly summarize their finding as part of the introduction. It is general practice now for introduction to include the summary of important findings in an article.
2. The authors run Probit and Tobit modesl: I don't know why they decided not to provide description of their model and variables in the paper, including assumptions made. A brief description of how their model is set up is necessary.

**Minor:**

1. There should be at least a footnote defining "Woreda" the first time it is mentioned on page 4.
2. Similarly, "kebeles" should be described briefly as it is first mentioned in page 5.
3. In table 1, under the "Characteristics of dairy farm households", the authors should provide information about the distribution of the variables. Something like standard deviation in parenthesis will be helpful.
4. Inconsistency in units labeling: In table 3; are mean and median units? The unit labeling should be consistence.
5. Table 5: "Travel time to access an A.I. agent, in minutes" - the unit is not consistent with the rest. The unit should be "Minutes" while variable should be labeled "Median travel time to access an A.I. agent."
6. Similarly, the unit under "Administrative data" should be corrected
7. On page 16: "livestock death rates declined over the last decade"; declined from what value to what value?
